# *Escherichia coli* Response to Uranyl Exposure at Low pH and Associated Protein Regulations

**DOI:** 10.1371/journal.pone.0089863

**Published:** 2014-02-26

**Authors:** Arbia Khemiri, Marie Carrière, Nicolas Bremond, Mohamed Amine Ben Mlouka, Laurent Coquet, Isabelle Llorens, Virginie Chapon, Thierry Jouenne, Pascal Cosette, Catherine Berthomieu

**Affiliations:** 1 CEA, DSV, IBEB, Commissariat à l'Energie Atomique, Laboratoire des Interactions Protéine-Métal, Saint-Paul-lez-Durance, France; 2 CNRS, UMR Biologie Végétale et Microbiologie Environnementales 7265, Saint-Paul-lez-Durance, France; 3 Université d'Aix-Marseille, Saint-Paul-lez-Durance, France; 4 UMR E3 CEA-Université Joseph Fourier, Service de Chimie Inorganique et Biologique, Laboratoire Lésions des Acides Nucléiques (LAN), Grenoble, France; 5 UMR 6270 CNRS, Plateforme Protéomique PISSARO, IRIB -Université de Rouen, Mont Saint Aignan, France; 6 ESRF-CRG-FAME beamline, Polygone Scientifique Louis Néel, Grenoble, France; 7 Commissariat à l'Energie Atomique CEA, DSM, INAC, Laboratoire Nanostructure et Rayonnement Synchrotron, Grenoble, France; Institut Pasteur, France

## Abstract

Better understanding of uranyl toxicity in bacteria is necessary to optimize strains for bioremediation purposes or for using bacteria as biodetectors for bioavailable uranyl. In this study, after different steps of optimization, *Escherichia coli*cells were exposed to uranyl at low pH to minimize uranyl precipitation and to increase its bioavailability. Bacteria were adapted to mid acidic pH before exposure to 50 or 80 µM uranyl acetate for two hours at pH≈3. To evaluate the impact of uranium, growth in these conditions were compared and the same rates of cells survival were observed in control and uranyl exposed cultures. Additionally, this impact was analyzedby two-dimensional differential gel electrophoresis proteomics to discover protein actors specifically present or accumulated in contact with uranium.Exposure to uranium resulted in differential accumulation of proteins associated with oxidative stress and in the accumulation of the NADH/quinone oxidoreductase WrbA. This FMN dependent protein performs obligate two-electron reduction of quinones, and may be involved in cells response to oxidative stress. Interestingly, this WrbA protein presents similarities with the chromate reductase from *E. coli*, which was shown to reduce uranyl *in vitro*.

## Introduction

Uranium is a heavy radioactive metal naturally present on earth, which has been widely exploited for industrial and military applications. Mining activities, uranium processing and leaching of wastes are significant anthropogenic sources of uranium dissemination in the environment, in subsurface sediments or ground water. Although metals are often met in biological systems and are essential for living organisms, uranium is universally recognized as a toxin, presenting both chemical and radiological toxicity to living organisms [1], [2].

In the environment, uranium is predominantly observed in two oxidation states, the oxidized U(VI) form, mainly represented as the uranyl UO_2_^2+^ cation in the presence of oxygen, and the reduced form U(IV), mainly occurring as insoluble species, stable in anoxic conditions. In addition, uranyl may be associated to a large range of organic or inorganic ligands as carboxylates, carbonates or phosphates [3], that determine its solubility and hence its bioavailability, i.e. its ability to bind to or traverse the cell surface of an organism. These properties -called uranium speciation- have been demonstrated to be both pH and concentration dependant [3], [4].

Interactions between uranium and bacteria have been extensively studied to identify the potential role of soil bacteria to change the speciation of uranium and its bioavailability. This work also led to evaluate their potential for bioremediation of contaminated areas [5], [6], [7], [8]. Besides uranium reduction in anoxic conditions, the most frequent phenomenon described for uranium-bacteria interaction is uranium biosorption or precipitation at cell surface. Uranium binding to cell envelopes (cell wall/membrane) involvescoordinationby negatively charged groups, such as carboxylates and phosphoryl groups [7], [9]–[13]. Precipitation as uranyl phosphate [11], [14], [15] or calcium-uranium-phosphate were notably documented [15], [16], [17], [18]. These interactions largely depend on the properties of cell wallsand of the external milieu and may be independent of cells viability [7].

There is far less information concerning intracellular uranium accumulation, or consequences of uranium exposure in bacterial cells [7]. Uranyl penetration in cells is thought to be associated with increased membrane permeability, and intracellular uranium sequestration considered as a passive process involving formation of inorganic compounds as polyphosphate granules [19], [20]. It is of major interest to enlarge our knowledge on the cellular response of cells to uranium exposure to better understand toxicity mechanisms of uranyl in bacteria and, as a consequence, to identify potential interesting mechanisms in terms of bioremediation.

The response to uranium exposure of environmental bacteria such as *Caulobacter crescentus* [16], *Geobacter uraniireducens* [21], and *Shewanella oneidensis* [22] was previously analysed at the transcription level. In the uranium tolerant species *C. crescentus*, response to uranium did not overlap substantially with other heavy metal stresses [16] and this specific response was used subsequently to develop a whole cell uranyl biosensor [23]. A proteogenomic approach was also conducted to analyse the evolution of *Geobacter* community structure and physiology under stimulated uranium reduction [24].

While several studies have used two-dimensional gel electrophoresis (2D–E) to investigate changes in protein expression of many bacteria after exposure to a wide range of toxic or biological metals, e.g., silver [25], copper, lead, cobalt [26], [27], [28], or chromium [27], [29], [30], none focused on the effect of uranium. This may be due to difficulties associated with the control of uranyl speciation in bacteria culture media. Only two papers described the impact of uranium stress on lung and kidney cells proteomes [31], [32], [33].

To help uncover the mechanisms of toxicity associated with exposure to uranyl, we developed an experimental model to expose the bacterial model *Escherichia coli* to uranium in conditions allowing uranium bioavailability and cells survival. To reach this objective, we challenged bacterial cells with uranium at low pH in a diluted LB medium supplemented with glucose.For these experiments, we took advantage of the acid resistance of *E. coli*, one of the best scientifically analyzed organisms that represents an invaluable tool in molecular microbiology and biotechnology.The uranium speciation in the exposure medium was analyzed by means of X-ray absorption spectroscopy (XAS), and we analyzed the proteomic response of the bacteria in light of its genome annotation.The regulated proteins were identified by mass spectrometry after two-dimensional gel electrophoresis (2D–E). The data that we report here may be of interest to optimise strains for biodetection or bioremediation purposes, notably in frame of optimizing in-cell sequestration of uranium.

## Results and Discussion

### 1. Uranium challenge conditions

Uranium precipitates as uranium phosphate in culture media at pH above 3.5–4.5 [34], limiting its bioavailability. Exposure of bacterial cells to uranyl has often been performed in water or water supplemented with 0.1 M NaCl or 0.1 M NaClO_4_ at various pH, including pH as low as 2 or 3, [7], [35] or in the presence of strong uranyl complexing agents as citrate [17].These exposure conditions were used to avoid uranyl precipitation with phosphate that could occur in rich growth medium, or in minimal mineral medium containing phosphates.In addition, a large number of bacteria precipitate uranium in the form of inorganic uranyl-phosphate complexes at the cell surface or in the medium at neutral to mid acidic pH [7]. In these conditions, low uranyl bioavailability impairs the analysis of the impact of moderate concentrations of uranyl on bacterial cells physiology.

With *Microbacterium* isolates, uranyl precipitates in the form of inorganic phosphate complexes (meta-autunite) at pH 4.5, while it interacts with organic phosphate groups at more acidic pH [35]. Interaction of uranyl with organic phosphate was also described at low pH for *Bacillus subtilis* [12], and with protonated phosphoryl groups and carboxylic sites for *Pseudomonas fluorescens* [36]. These data are in line with a higher bioavailability of uranyl at low pH.

**Figure 1:**
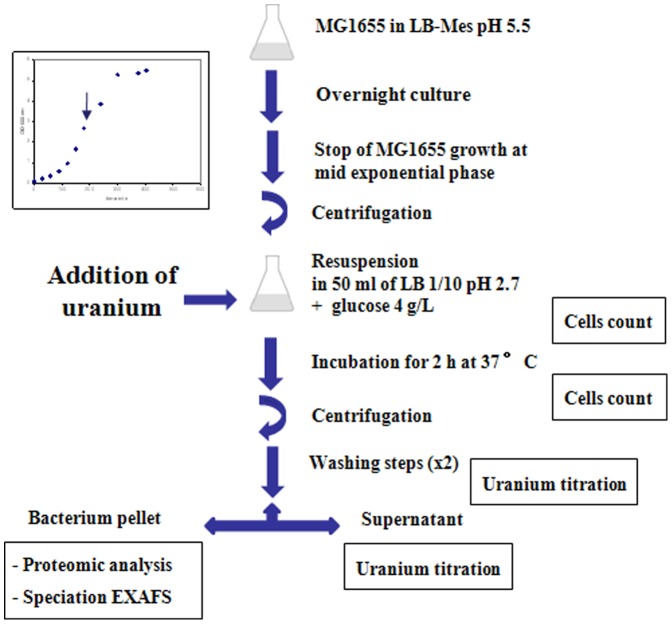
Schematic view of *E. coli* cells exposure to uranium and details of the optimized analysis protocol.

In this study, we took advantage of the acid resistance property of *E. coli* to characterize the *E. coli* K-12 strain MG1655after uranium 238 (^238^U) exposure in LB (1/10)-glucose medium at pH 2.7 (Figure 1). These experimental conditions of exposure were chosen mainly for two reasons. First, the pH was fixed below pH 4 and the LB medium diluted tenfold to minimize the level of complexation between uranyl and phosphate orcarbonate groups. In addition, diluted LB supplemented with glucose was chosen for the presence of various amino acids and organic molecules since this may reduce the level of stress engendered by the hostile growth conditions (i.e. low pH anduranyl exposure). Second, *E. coli* has been used in basic research as a model organism for a long time. Physiology and genetics of this bacterium are among the best characterized of all species. Accordingly, the activity of its encoded proteins is probably at the best level of understanding in bacteria.

### 2. Uranyl speciation in the exposure medium

**Figure 2:**
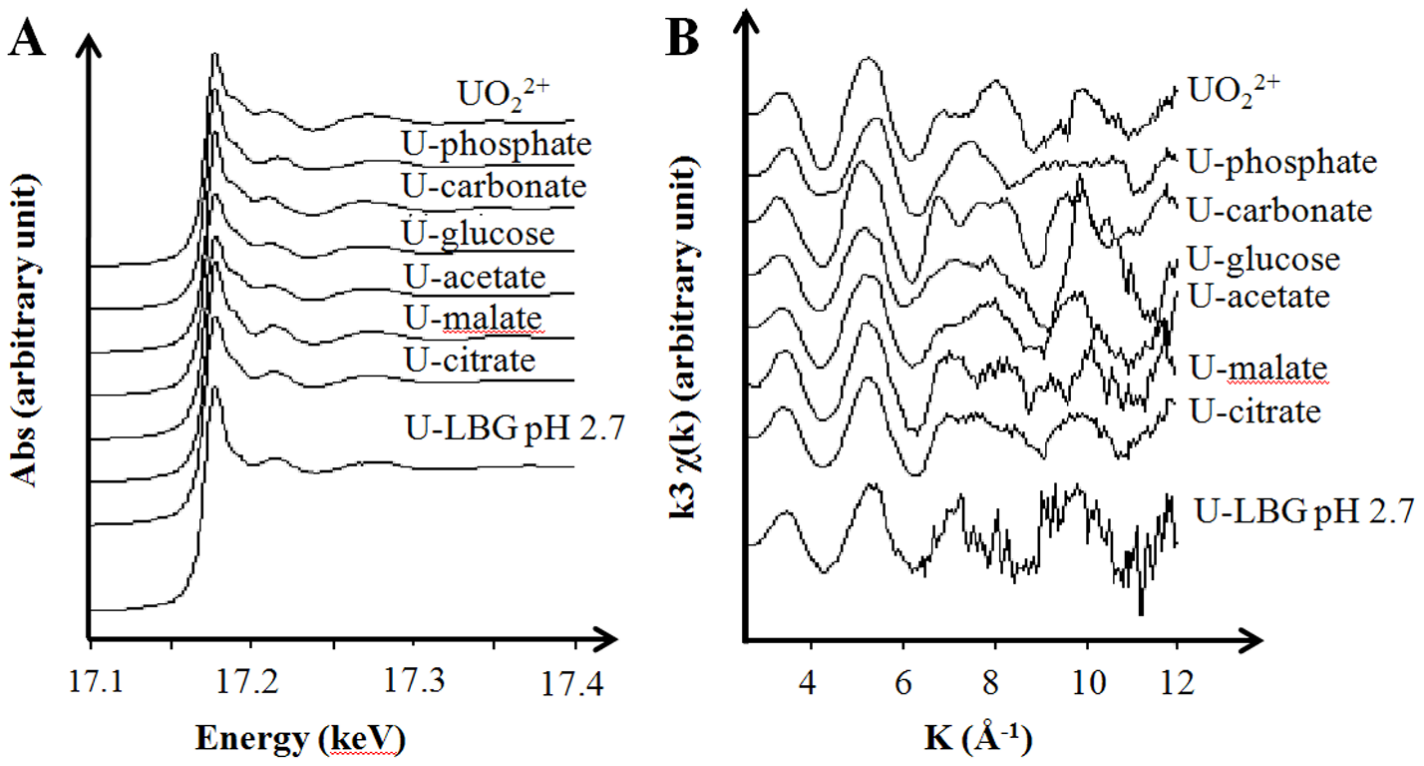
Uranium speciation in the exposure medium. Uranium L_III_-edge XAS spectra (A), normalized to equal intensity at 17.176 keV; and k^3^-weighted EXAFS curves (B) of uranyl acetate 50 µM prepared in LB-glucose medium at pH 2.7 (U-LBG pH 2,7) compared to reference spectra.

The speciation of uranyl in the exposure medium was analyzed using X-ray absorption spectroscopy (XAS) (Figure 2). This medium, dedicated to bacterial growth is mainly composed of proteins or amino acids, sugar moieties and salts. It contains phosphate residues, but the presence of strong uranyl ligands such as carbonate or citrate is unexpected. Consequently, uranyl may be found as complexes with phosphate, glucose, or carboxylate moieties of proteins and amino acids residues [37], [38]. Given the complexity of this medium, uranium speciation is suspected to be rather complex.

To get a more precise picture of uranium complexation, a XAS spectrum of the exposure medium (diluted LB medium with glucose at pH 2.7, see below) supplemented with 50 µM uranyl acetate was recorded at the uranium L_III_-edge, and compared to spectra of reference uranyl complexes (Figure 2). The exposure medium was analyzed in the same conditions as during bacterial exposure even if the uranyl concentration is close to the limit of detection of the majority of synchrotron beamlines (including those dedicated to the analysis of diluted samples such as FAME: BM30B, ESRF, France). However, changing the uranyl concentration in the medium would have modified its speciation.

In these conditions, the acquired spectra are noisy and their analysis can only be partial. The XANES (X-ray Absorption Near Edge Structure) region and the position of the absorption edge (∼17.177 keV, Figure 2-A) is however characteristic of the U(VI) oxidation state [39]. The shoulder at 17.190 keV after the absorption edge, typical from the linear uranyl dioxocation UO_2_^2+^, is absent. This suggests that this linear structure may be distorted in the uranyl complexes formed in the medium. In addition, EXAFS spectra (Extended X-ray absorption fine structure Figure 2-B) show a first peak massif (4.5–6 Å^−1^) with its maximum aligned with that of spectra recorded with UO_2_^2+^ or U-malate, U-acetate, or U-glucose complexes, but not with U-phosphate or U-carbonate complexes. The remaining of the EXAFS spectrum, although very noisy, also differs from spectra recorded with U-phosphate and U-carbonate complexes. The second peak massif (6.5–8.8 Å^−1^), aligns with contributions present in the U-malate spectrum, while the peak massif at 9–11 Å^−1^ has a strong contribution in the U-glucose spectrum. Even if this interpretation is strictly qualitative, uranium speciation in the LB-glucose medium may be a mixture of glucose and carboxylate complexes of uranyl. Therefore uranyl is expected to be bioavailable for *E. coli* cells in these experimental conditions.

### 3. Exposure assay of *E. coli* cells

Acid resistance is an inherent property of the enteric bacterium *E. coli*, associated to its necessity to develop strategies to overcome acidic gastric conditions [40]. In addition to acid resistance associated with the stationary phase, situations of tolerance have also been described in the exponential growth phase [41], [42]. Moreover, the survivability of *E. coli* cells has been reported at a pH as low as pH 2.5 after preculture in a moderate acidic medium overnight.

**Figure 3:**
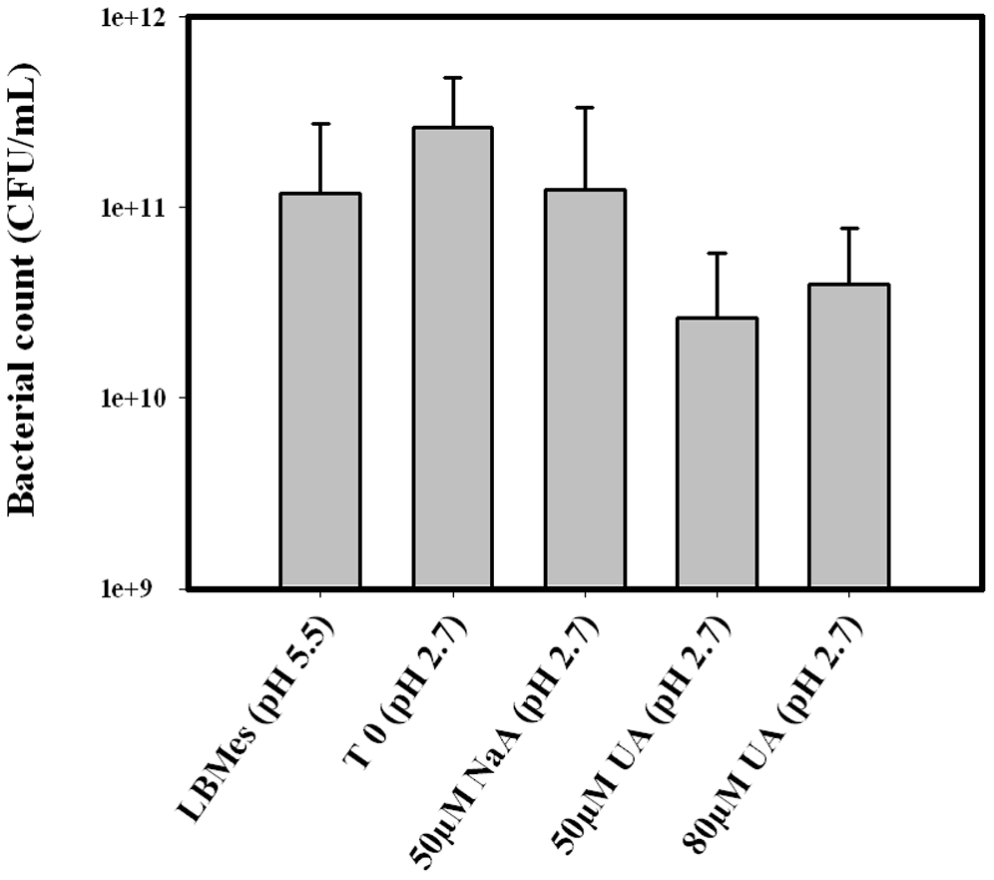
Cell survival after exposure to uranyl at acidic pH. Numbering of Colony Forming Units from the *E. coli* culture in LB-Mes pH 5.5, and in the exposure LB 1/10 Glucose medium at pH 2.7 before (T0) and after exposure for 2 hours to 50 µM sodium acetate, 50 µM of uranyl acetate, and 80 µM of uranyl acetate.

In this study, *E. coli* cells were grown in LB-Mes medium at pH 5.5 to promote resistance to mild acid conditions and then exposed to uranyl at pH 2.7 for two hours. As summarized in Figure 1, a LB-Mes pH 5.5 medium was inoculated at 1/100 (v/v) with an overnight culture grown in LB-Mes pH 5.5, and the cells grown to mid exponential phase. Cells were then collected by centrifugation and exposed at pH 2.7 in a tenfold diluted LB medium supplemented with glucose (4 g/L) and 50 µM sodium acetate (control samples) or uranyl acetate (exposed samples) at 50 µM or 80 µM concentrations. The rate of cells survival after the uranium challenge was evaluated by numeration of colony forming units (CFU). These data showed that exposure of *E. coli* cells for two hours at pH 2.7 (medium: LB 1/10 + glucose) resulted in around 15% cell viability (Figure 3). More interestingly, similar percentages of cell survival were obtained for control samples and samples exposed to 50 µM or 80 µM of uranyl acetate.

The cells were then harvested by centrifugation, washed twice in Tris buffer (pH 7), and the uranyl concentration in the exposure media and post-exposure supernatants was measured using UV-visible spectrophotometry [43]. Less than 5% of the total uranium was still soluble in the exposure medium after the 2 hours exposure of *E. coli* cells. About 25% to 30% of the uranium was further released by the washing steps in Tris buffer (pH 7), reflecting uranium weakly associated to the bacterial cells. Consequently, we deduced percentages of 73 and 65% of the uranium were retained in the cell pellets after 2 h of cells exposure to 50 µM or 80 µM of uranyl acetate, respectively (Table 1). These measurements demonstrated that a main fraction of the uranium was retained by bacteria.

**Table 1:** Uranyl concentrations in the supernatants and in the cell pellets.

	Control	50 µM	80 µM
Uranyl in supernatant after 2 hours exposure at pH 2.7 (%)	0	2.4±1.8	4.1±2.2
Uranyl fraction released upon Tris washing (%)	0	24±8	31±2
Uranyl present in the cell pellets (deduced) (%)	0	≈ 73%	≈ 65%

### 4. Proteomic response to uranyl exposure

**Figure 4:**
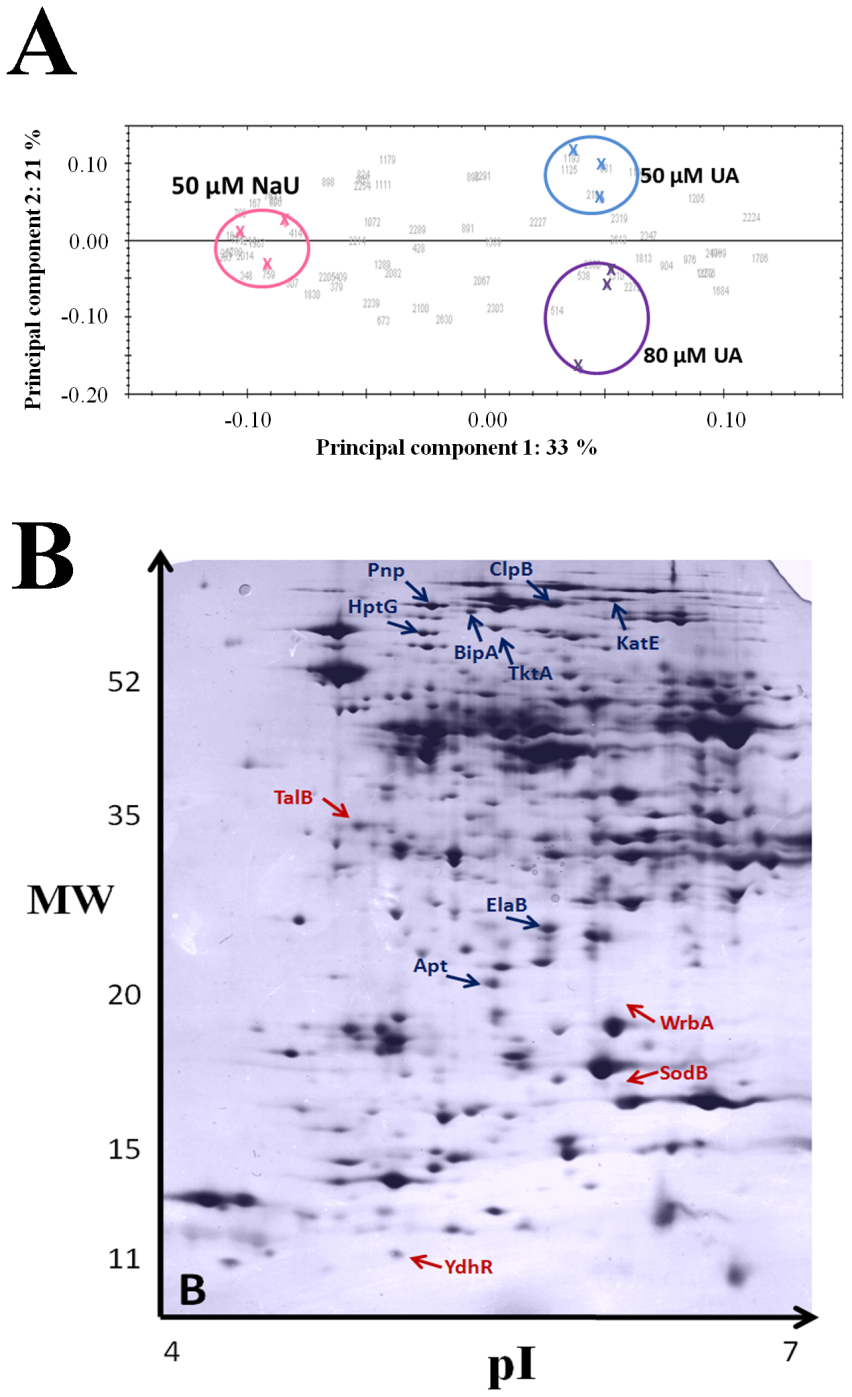
Protein regulations from 2D-gel analysis. A: Principal component analysis performed on the proteomes of control cells and cells exposed to 50 and 80 µM of uranyl acetate. Each colored cross represents one independent replicate. The numbers in grey represent the protein spots which are impacted by the uranium stress in a statistically pertinent manner. B: Two-dimensional gel-electrophoresis obtained with a control sample (2 hours sodium acetate), showing the position of up-regulated proteins (red circles) and down-regulated proteins (blue circles) in uranyl exposed cells (protein loading: 100 µg; silver nitrate staining).

The proteomic response to uranyl exposure was investigated on three independent experiments, where samples exposed to 50 or 80 µM of uranyl acetate were compared to control samples exposed to 50 µM sodium acetate. After protein spots abundances measurements, PCA analysis was performed on normalized data (vertically and horizontally, Figure 4A). It revealed that the proteome of control samples is significantly different from that of *E. coli* cells exposed to 50 or 80 µM of uranyl acetate. The first component, which explains more than 33% of the total variability, strongly discriminates the uranyl exposed samples with respect to the control ones, arguing for the physiological impact of the uranium treatment. The second component, accounting for 21% of the total variability, separates the samples exposed to 50 µM and 80 µM uranyl acetate, indicating here the concentration-dependence of the bacterial response. Those results show clearly an effect of uranium on the bacterial proteome and also further evidence its bioavailability for cells in the optimized experimental culture conditions.

At the protein level, eleven spots were differentially regulated in uranyl exposed cells, i.e., fulfilling the described criteria (see Methods). These spots were identified by mass spectrometry (Table 2). The corresponding proteins were observed in a large range of pI and protein masses (as illustrated in Figure 4B). Two protein groups were discriminated, according to their behavior, i.e., up or down-regulation after uranyl acetate exposure (Table 2).

**Table 2:** Proteins with differentiated abundances in uranyl exposed *E. coli* cells.

Protein	MW Da	pI	S	Np	%	Name	Code	Norm V±SD			Fold 50 UA/50 NaA	p-value	Fold 80 UA/50 NaA	p-value
								50 µM NaA	50 µM UA	80 µM UA				
** Up-regulated**														
TrpR binding protein WrbA	20832	5.59	159	5	35	WrbA	b1004	3.8 10^5^±5.0 10^4^	2.5 10^6^±1.3 10^6^	3.4 10^6^±1.2 10^6^	6.64	0.04	9.08	0.01
Superoxide dismutase. Fe	21253	5.58	122	5	22	SodB	b1656	1.4 10^6^±5.1 10^5^	7.1 10^6^±6.4 10^6^	1.0 10^7^±4.7 10^6^	-	NS	7.18	0.03
Hypothetical protein b1667	11281	5.09	118	4	34	YdhR	b1667	2.4 10^6^±5.8 10^5^	4.8 10^6^±1.8 10^6^	8.1 10^6^±4.1 10^6^	-	NS	3.39	0.05
Transaldolase B	35197	5.11	79	3	10	TalB	b0008	1.7 10^6^±1.6 10^5^	2.7 10^6^±9.7 10^5^	4.7 10^6^±6.7 10^5^	-	NS	2.78	0.002
** Down-regulated**														
Polynucleotide phosphorylase/polyadenylase	79797	5.39	147	6	10	Pnp	b3164	2.6 10^6^±8.9 10^5^	4.6 10^5^±1.6 10^5^	1.6 10^6^±4.7 10^5^	5.55	0.01	-	NS
Adeninephosphoribosyltransferase	19847	5.26	81	2	16	Apt	b0469	1.9 10^6^±6.4 10^5^	3.5 10^5^±2.3 10^5^	5.0 10^5^±4.6 10^5^	5.26	0.02	4.54	0.04
Hydroperoxidase HPII(III) (catalase)	84140	5.54	57	2	1	KatE	b1732	1.6 10^6^±4.1 10^5^	7.3 10^5^±5.5 10^5^	3.5 10^5^±2.5 10^5^	-	NS	4.54	0.01
GTP-bindingprotein	67313	5.16	40	2	4	BipA	b3871	8.7 10^5^±1.9 10^5^	2.1 10^5^±7.0 10^4^	3.8 10^5^±2.3 10^5^	4.17	0.004	-	NS
Hypothetical protein b2266	11299	5.35	62	2	24	ElaB	b2266	2.9 10^6^±1.3 10^6^	7.4 10^5^±4.5 10^5^	1.0 10^6^±7.4 10^5^	4.00	0.05	-	NS
Molecular chaperone with ATPase activity	71378	5.09	217	7	16	HtpG	b0473	1.6 10^6^±7.0 10^5^	4.9 10^5^±1.9 10^5^	1.3 10^6^±4.5 10^5^	3.33	0.05	-	NS
Protein disaggregation chaperone	95526	5.37	354	12	19	ClpB	b2592	1.76 10^6^±1.9 10^5^	5.9 10^5^±2.3 10^5^	8.1 10^5^±6.2 10^4^	3.03	0.002	2.17	0.001

MW: Molecular weight; pI: Isoelectric points; S: Identification score; Np: Number of identified peptides; %: Sequence coverage from identification results; Code: NCBI entry; NormV±SD: Averaged normalized spot volume±standard deviation; NaA: sodium acetate; UA: uranyl acetate; NS is indicated when the fold change was not significant.

#### 4.1. Oxidative stress

Exposure to 80 µM uranyl acetatesignificantly affected the cells content in proteinsinvolved in oxidative stress response SodB and KatE. Fe-superoxide dismutase SodB was accumulated by more than 7 fold after exposure to 80 µM of uranyl acetate (Table 2). SodB is associated with the elimination of superoxide ions in the cytoplasm, by conversion into oxygen and hydrogen peroxide. In contrast, the hydroperoxidase HPII(III) (catalase) KatE was less abundant by a factor of 4.5 in cells exposed to 80 µM of uranyl acetate. KatE catalyzes the dismutation of hydrogen peroxide into water and oxygen. The increased SodB content in the presence of uranyl may suggest that uranyl exposure induces an oxidative stress mediated by the formation of superoxide radicals rather than by the formation of hydrogen peroxide.The strong up regulation of SodB in *E. coli* cells exposed to uranyl contrasts with transcriptomic results reported on uranyl exposure of *C. crescentus* cells [16]. In this study, no change in the expression of the *sodB* gene was observed, but the gene coding for Mn superoxide dismutase, SodA, was up-regulated by a factor of 2.9, upon *C.crescentus* cells exposure to 200 µM of uranyl nitrate [15]. These different superoxide dismutases, associated with different metal binding: Mn for SodA and Fe for SodB, exert a similar function of superoxide detoxification.

#### 4.2. Redox reactions associated to oxidative stress

The most largely up-regulated protein upon uranium exposure, WrbA, is an oligomeric protein, that binds one molecule of flavin mononucleotide (FMN) per monomer [44]. This protein was respectively accumulated by factors of 6.64 and 9.08 in cells exposed to 50 µM and 80 of µMuranyl acetate. WrbA was characterized as well as under control of the master regulator RpoS [45], [46] and as a prototype of FMN containing NAD(P)H/quinone oxidoreductase (NQO) and classified as a type IV NQO protein [47]. It was shown to perform obligate two-electron reduction of quinones [47], a process that may be involved in the protection of cells against oxidative stress, by avoiding the reaction of semiquinone forms with oxygen. In addition to WrbA, a conserved hypothetical protein YdhR was also accumulated ≈3.4 fold in cells exposed to 80 µM of uranyl acetate (Table 2). YdhR is yet uncharacterized, however proteogenomic analysis revealed that YdhR (b1667) likely belongs to an identified group of mono-oxygenases (including ActVA-Orf6 and YgiN) involved in the oxygenation of polyaromatic ring compounds [48].

Interestingly, WrbA bears large similarity with other FMN-containing NAD(P)H/quinone oxidoreductases, as the chromate reductase (ChrR), which was previously shown to reduce a large range of molecules including chromate [49] or uranyl *in vitro* [50]. The ability to reduce chromate is shared by several FMN containing proteins of the flavodoxin family. However, it was observed that differences exist between these proteins concerning their ability/efficiency to perform the two-electron reduction of these compounds [51]. In particular the chromate reductases from *E. coli* and *Pseudomonas putida* were shown to differ in the rate of ROS production upon chromate reduction [49].

#### 4.3. Additional stress responses

Exposure of *E. coli* cells to 50 and 80 µM of uranyl acetate led to the strong down-regulation of adenine phosphoribosyl transferase (Apt), that is involved in the synthesis of AMP from adenine and ribosylpyrophosphate. The polynucleotidephosphorylase (Pnp) was down regulated in cells exposed to 50 µM of uranyl acetate. Pnp is an exoribonuclease primary involved in single-stranded RNA degradation and is part of the RNA degradosome [52]. It was recently shown, that the stability of several small RNAs decreased in Δ*pnp* mutants [53], which affected their regulatory function.

Finally, two heat shock proteins, the protein disaggregation chaperone ClpB and the molecular chaperone HtpG (Hsp90) are significantly less abundant in cells exposed to 50 µM of uranyl acetate than in control cells. This behaviour is unclear since these two heat shock proteins are chaperones involved in the stabilisation of proteins and have been shown to facilitate the folding of newly synthesized proteins in *E. coli* [54].

### Concluding remarks

The experimental conditions used to challenge *E. coli* cells at low pH allowed the analysis of the proteomic response to uranyl exposure in conditions in which uranyl is associated with weak ligands as glucose and/or organic compounds and thus bioavailable to cells. Although, only 15% of cell survival was obtained after the 2 hours challenge at acidic pH, similar survival rates were obtained for both control and exposed cells, allowing to record reliable comparative proteomic data. Indeed, the PCA analysis showed a clear separation of exposed and control cells.

These proteomic data suggest that uranyl exposure in these conditions generates an oxidative stress probably due to redox reactions directly or indirectly mediated by uranyl. These results are supported by the up-regulation of WrbA and YdhR, possibly involved in a quinone redox cycling mechanisms. Since WrbA presents sequence homology with the chromate reductase of *E. coli* which was demonstrated to reduce uranyl *in vitro* by a “*safe*” two electron reduction mechanisms [49], [55], it would be interesting to study whether WrbA may be directly involved in uranyl reduction.

## Methods

### 1. Bacterial strain

This *E. coli* strain MG1655 was used. Bacteria were maintained as glycerol stocks and stored at −80°C.

### 2. Growth conditions

Overnight pre-cultures in 20 mL of LB-Mes 100 mM pH 5,5 medium were inoculated with single colonies from LB agar plates and incubated at 37°C under agitation (150 rpm). The cultures were diluted in 100 mL of LB-Mes medium (Luria-Bertani medium LB buffered with 100 mM MES (morpholineethanesulfonic acid) pH 5.5) and incubated at 37°C under agitation (150 rpm). The bacterial growth was followed by measuring the optical density at 600 nm (OD). When the mid exponential growth phase was reached (i.e., OD_600_ = 0.6), cells were collected by centrifugation at 4000× g for 10 min and resuspended in 50 ml of LB medium diluted at 1/10 at pH 2.7 and supplemented with 4 g/L of glucose. In each flask, 50 µM or 80 µM of uranyl acetate or sodium acetate as control, were added. The cultures were exposed for 2 hours at 37°C under agitation. Finally each bacterial culture was harvested by centrifugation at 4000× g, for 10 min at 4°C, and the pellet was washed twice in 50 ml of Tris 100 mM at pH 7.

### 3. Uranium speciation using XAS

XAS experiments were performed at the U L_III_-edge (17.166 keV) on the BM30B beamline[56] of the European Synchrotron Radiation Facility, Grenoble, France. The storage ring was operated in 16 bunches mode at 6 GeV with a ∼90 mA current. The beam energy was selected using a Si(220) double-crystal monochromator with an experimental resolution close to that theoretically predicted (∼0.4 eV). The beam size on the sample was approximately 300×200 µm (H x V). Spectra were recorded in fluorescence mode for the samples and liquid references, using a 30-element solid state Ge detector (Canberra, St Quentin en Yvelines, France), and in transmission mode for one solid sample, pressed as a pellet (U-phosphate).

Spectra were normalized and EXAFS oscillations were extracted using the Athena code [57]. The resulting EXAFS curves were weighted by k^3^ and qualitatively analyzed.

### 4. Dosage of uranium and bacterial enumeration

The uranium concentration in the supernatant was determined by spectrophotometric measurements using 2-(2-Thiazolylazo)-p-Cresol (TAC), as described in [43]. Bacterial survival rates were determined by counting bacterial colonies plated on LB agar medium at varying dilution rates before and after incubation with 50 µM or 80 µM of uranyl acetate or sodium acetate at pH 2.7.

### 5. Protein extraction and solubilisation

After centrifugation, the bacterial pellet was freeze-dried for 2 h and re-suspended in isoelectric focalisation (IEF) buffer of the following composition: 7 M urea, 2 M thiourea, 0.1% amidosulfobetaine (ASB14), 20 mM Dithiotheritol (DTT), 2% (v/v) carrier ampholytes (pH 4–7; GE Healthcare). The mixture was incubated twice during 10 min successively at 37°C and −80°C, and subsequently submitted to two cycles of 1 min sonication on ice. Protein extracts were then harvested in the supernatant after elimination of cellular debris by centrifugation (10,000× g for 30 min). Protein concentrations were evaluated using the Bio-Rad protein assay and protein extracts were stored at −20°C until further analysis.

### 6. Isoelectric focusing (IEF)

For IEF, an additional volume of IEF buffer was added to the protein solution containing 100 µg of proteins in a final volume of 400 µl (with 0.4% of Coomassie blue). IEF was carried out with immobilised pH gradients (Immobiline DryStrips pH 4–7, 18 cm NL GE Healthcare). After passive rehydration for 24 h, IEF was performed by using a Multiphor II (GE Healthcare) as follows: 150 V for 1 h, 350 V for 15 min, 750 V for 45 min, 1.5 kV for 1 h, and 3.5 kV for 17 h (1 mA, constant) for a total of 70 kVh.

### 7. SDS-PAGE

After two equilibration steps dedicated to reduction/alkylation of cysteins (using DTT (25 mM) and iodoacetamide (50 mM) respectively), the second dimension was obtained by SDS-PAGE, carried out with a Protean® II xi cell (Bio-Rad), and using a 12.5% (w/v) polyacrylamide resolving gel (width, 16 cm; length, 20 cm; thickness, 0.75 mm). The gels were fixed overnight and proteins were revealed using the silver nitrate staining. Gels were stored in water at 4°C before spot excision. For each condition, three independent cultures were obtained and the corresponding gels realized in triplicate.

### 8. Statistical analysis

Gels were scanned using the Fluorimager ProXpress from PerkinElmer and analyzed using the SameSpots 4.0 software (Nonlinear Dynamics, Newcastle upon Tyne, UK). Images were primarily tested for similar dynamic range and absence of saturation, and entirely pre-aligned before detection, resulting in optimal matching between all gels. The statistical tool integrated into the software, combines calculation of *p* values (ANOVA), *q* values, power, correlation analysis and Principal Component Analysis (PCA). PCA was used to interpret spot quantity variation among the different experimental conditions tested. This analysis is based upon the generation of a covariance matrix describing how each spot varies with respect to every other spot in the data set.

For this analysis, ANOVA was calculated using a variance stabilisation technique based on the logarithm of normalised volume. The spots with *p* value <0.05 and a power >0.8 were selected. In addition to statistical parameters a 2-fold ratio for significant spot alteration has been arbitrarily chosen. In order to remove false positives, spots with *q* values >0.15 were rejected [58].

### 9. Protein identification

#### 9.1. Tryptic digestion

After protein spots excision from 2D gels, the excised fragments were washed several times and dried in a SpeedVac centrifuge for few minutes. Trypsin digestion (with 10 µL of a 10 ng/µL trypsin solution) was performed overnight with a dedicated automate (MultiPROBE II, Perkin Elmer). Thereafter, gel fragments were subsequently incubated twice for 15 min in a H_2_O/CH_3_CN (1∶1) solution to extract the peptides from the gel. The peptide extracts were then dried and solubilised in starting buffer for chromatographic elution, consisting of CH_3_CN 3%/HCOOH 0.1% in water.

#### 9.2. Tandem mass spectrometry

The peptides were enriched and separated by reversed-phase LC with a precolumn/analytical column nano-flow setup (HPLC-Chip cube; Agilent Technologies). The peptides were further fragmented after a full survey scan (m/z 300-200) using an on-line ion trap mass spectrometer (model 6330, Agilent). MS/MS experiments of the 5 most abundant precursor ions were acquired and the fragmentation data were exported using the DataAnalysis Software (version 3.4, Bruker Daltonic).

#### 9.3. Analysis of peptide sequences

For protein identification, extracted MS/MS peak lists were compared to the *E. coli* ORF protein database using the MASCOT Daemon (version 2.1.3) search engine. All searches were performed with no fixed modification and with variable modifications for carbamidomethylation of cysteines and for oxidation of methionines, and with a maximum of one missed cleavage. MS^2^ spectra were searched with a mass tolerance of 1.6 Da for precursor ions and 0.6 Da for fragment ions, respectively. The protein identification was validated if 2 peptides exhibited fragmentation profile score higher than the average default value for significance using MASCOT.
